# The assessment and detection rate of intrinsic capacity deficits among older adults: a systematic review and meta-analysis

**DOI:** 10.1186/s12877-024-05088-w

**Published:** 2024-06-03

**Authors:** Fangqin Tan, Xiaoxia Wei, Ji Zhang, Yihao Zhao, Xunliang Tong, Jean-Pierre Michel, Ruitai Shao, Enying Gong

**Affiliations:** 1 School of Population Medicine and Public Health, Chinese Academy of Medical Sciences & Peking Union Medical College, 31, Beijige 3 Aly, Dongcheng District, Beijing, China; 2 Department of Pulmonary and Critical Care Medicine, Beijing Hospital, National Centre of Gerontology, Institute of Geriatric Medicine, Chinese Academy of Medical Sciences, Beijing, China; 3 University of Geneva, Geneva, Switzerland; 4 French Academy of Medicine, Paris, France; 5 State Key Laboratory of Respiratory Health and Multimorbidity, Chinese Academy of Medical Sciences & Peking Union Medical College, 31, Beijige 3 Aly, Dongcheng District, Beijing, China

**Keywords:** Intrinsic capacity, Older adults, Prevalence, Meta-analysis, Healthy ageing

## Abstract

**Background:**

Assessing and monitoring intrinsic capacity (IC) is an effective strategy to promote healthy ageing by intervening early in high-risk populations. This review systematically analyzed the global detection rates of IC deficits and explored variations across diverse populations and data collection methods.

**Methods:**

This study was preregistered with PROSPERO, CRD42023477315. In this systematic review and meta-analysis, we systematically searched ten databases from January 2015 to October 2023, for peer-reviewed, observational studies or baseline survey of trials that assessed IC deficits among older adults aged 50 and above globally following the condition, context and population approach. The main outcome was intrinsic capacity deficits which could be assessed by any tools. Meta-analyses were performed by a random-effect model to pool the detection rates across studies and subgroup analyses were conducted by populations and data collection methods.

**Results:**

Fifty-six studies conducted in 13 countries were included in the review and 44 studies with detection rates of IC were included in the meta-analysis. The pooled detection rate of IC deficits was 72.0% (65.2%-78.8%) and deficits were most detected in sensory (49.3%), followed by locomotion (40.0%), cognition (33.1%), psychology (21.9%), and vitality (20.1%). Variations in detection rates of IC deficits were observed across studies, with higher rates observed in low- and middle-income countries (74.0%) and hyper-aged societies (85.0%). Study population and measurement tools also explained the high heterogeneity across studies.

**Conclusion:**

IC deficits are common among older adults, while heterogeneity exists across populations and by measurement. Early monitoring with standardized tools and early intervention on specific subdomains of IC deficits are greatly needed for effective strategies to promote healthy ageing.

**Supplementary Information:**

The online version contains supplementary material available at 10.1186/s12877-024-05088-w.

## Background

Population ageing is a rising global health challenge and an undeniable demographic shift that affects numerous countries. A recent projection indicates a substantial increase in the proportion of individuals aged 60 or above globally, rising from 12% in 2015 to 22% in 2050 [1]. The speed of the demographic shift to an aged society is particularly rapid in some low- and middle-income countries (LMICs) [2]. Such demographic shifts have profound implications for public health and healthcare systems, underscoring the pressing need to implement effective strategies to promote healthy ageing [3].

Healthy ageing was defined by the World Health Organization (WHO) as the process of developing and maintaining the functional ability that enables well-being in older age [1]. Intrinsic capacity (IC) refers to the physical and mental attributes and abilities that an individual possesses throughout their life course. It serves as the core of healthy ageing, and interacts with relevant environmental characteristics to determine individuals’ functional ability [1, 4]. IC encompasses a range of physical and mental functions necessary for well-being and independent living, covering five subdomains, including cognition, locomotion, vitality, psychology, and sensory capacity (vision and hearing) [4, 5]. According to the existing literature, IC could also serve as a predictive measure for adverse health outcomes among older adults, such as the decline of functional ability, compromised activities of daily living, and the onset of frailty [6]. Thus, capturing the deficits of IC plays a pivotal role in implementing early intervention and promoting healthy ageing, which also reflects the concept of transitioning from a disease-centered to a function-centered approach in elderly care [7, 8].

Since the publication of the Integrated Care for Older People (ICOPE) in 2017 [9], which focused on assessing and improving IC to help older individuals maintain functional abilities, a number of studies have been conducted to identify individuals with IC deficits [10–13]. Literature also suggests that such assessment and monitoring could inform individuals’ trajectory in health, triage individuals with high risk of frailty, and offer opportunities for early intervention [7]. A few studies also piloted the implementation of ICOPE in multiple countries by using the ICOPE two-step tools for screening and in-depth assessment of individuals with IC deficits [10, 11, 13]. A few systematic reviews have synthesized findings from studies that focused on IC, by emphasizing the definition of IC, the tools used for IC measurement across studies and the detection of IC deficits [14–16]. However, these reviews were limited to studies that employed certain tools, such as ICOPE tools, for assessing IC, or were limited to certain countries only [16]. There is a general lack of comprehensive synthesis of evidence on how IC was assessed across studies, the detection rates of IC deficits across populations, data collection methods, and factors associated with IC deficits.

In response to this research gap, our study aims to perform a comprehensive review of international studies that assessed IC without imposing restrictions on the choice of IC measurement tools, to quantify the detection rates of deficits in IC and its subdomains, and to synthesize findings on factors associated with IC deficits. The evidence generated from this study will provide a global snapshot of IC deficits among older adults, which may help quantify the significance of the problem and highlight the importance of IC assessment and early interventions to promote healthy ageing.

## Methods

In this systematic review, we applied the Condition, Context, and Population (CoCoPop) framework to identify fundamental concepts relevant to the research questions, guide the development of the search strategies, and formulate the inclusion criteria for screening [17]. We focused on IC as the condition of interest, covering studies conducted in diverse settings globally, and included studies that assessed IC and examined IC deficits among middle-aged to oldest old populations. To enhance transparency and adhere to the best practices, this review was conducted by following Preferred Reporting Items for Systematic Reviews and Meta-Analyses (PRISMA statement) [18] and was registered at the International Prospective Register of Systematic Reviews, PROSPERO (CRD42023477315).

### Data sources and search strategy

To identify relevant studies, a systematic search was conducted in ten databases, including six databases in English (Pubmed, Embase, Web of Science, the Cochrane Library, PsychlNFO, and CINAHL) and four databases in Chinese (China National Knowledge Infrastructure, Wanfang database, Weipu database, and Sinomed). Following the principle of CoCoPop [17], we used terms pertinent to older people, intrinsic capacity, subdomains of intrinsic capacity, and ICOPE to generate the search strategies. The detailed search strategy for each database is provided in Additional file 1. The time frame for database searches spanned from January 2015, when WHO proposed the concept of intrinsic capacity, to October 2023.

### Criteria for inclusion and exclusion of studies

Following the CoCoPop framework, we set a series of inclusion and exclusion criteria. Studies were included if studies (i) reported the detection rates of deficits in IC or its subdomains or provided adequate data for calculation; (ii) were observational studies (including cross-sectional surveys, cohort studies, and case–control studies) or baseline surveys of trials. The exclusion criteria included: (i) not measured IC from five subdomains; (ii) secondary data analysis with duplicate findings from the same original study; (iii) non-original studies, such as conference abstracts, literature reviews, case reports, editorials, commentaries, etc.; (iv) articles written in a language other than English and Chinese; (v) articles for which full-text access was not available.

### Study selection and data extraction

All identified articles from the search were imported into Endnote v20 with duplicates removed. Two independent researchers (FT and XW) reviewed titles and abstracts, then assessed eligibility of the full text. Any disagreements were discussed with the senior reviewer (EG) until reaching a consensus.

A standard data extraction form was developed in a Microsoft Excel spreadsheet to gain detailed information from the eligible studies. The following information was extracted from all eligible studies: study information (title, author, year of publication), country of study (country name, economic status of countries, stage of ageing society of countries), study design (cross-sectional study, cohort study or baseline survey of trial), participants (sample size, inclusion criteria, percentage of female, mean age), data collection methods (settings of data collection, IC measurement tools), secondary data analysis (yes or no), key findings (detection rates of deficits in IC and its subdomains, associated factors or outcomes of IC deficits). The economic status of countries was classified according to the World Bank Classification [19]. We used data from World Population Prospects 2022 and applied WHO definition to classified countries into aging society (proportion of population aged 65 and above ≥ 7% of total population), aged society (≥ 14%) and hyper-aged society (≥ 21%) [20, 21]. Specifically, following previous studies [16], we defined the IC deficits as the presence of a decline in one or more subdomains of IC.

### Assessment of study quality

To evaluate the quality of studies, two independent researchers (FT and XW) assessed the eligible studies by using the Joanna Briggs Institute (JBI) critical appraisal tool for studies reporting prevalence data [17, 22]. This tool consists of nine items to evaluate the methodological quality of the observational studies that examine the prevalence of certain condition and has been widely applied to identify possible biases in study design, data collection, and data analysis.

### Statistical analysis

The statistical analysis was performed using Stata 17.0 [23] based on data extracted from the original studies. The detection rates of IC deficits were either obtained directly from the articles or calculated based on the available data extracted from the article. Cochran’s Q and the *I*^2^ statistic were used to assess whether there was significant heterogeneity among the studies [24]. Due to the diverse measurement tools of IC, as well as variations in population demographics, sample sizes, study settings, and designs, a high level of heterogeneity was expected (*I*^2^ = 99.9%). Accordingly, a random-effects model was employed to pool the detection rates of IC deficits [25]. The potential publication bias was assessed through visual funnel plots and Egger’s test [26]. In addition, we conducted subgroup analyses by utilizing random-effects model. Studies were classified by countries' characteristics, data collection settings, and IC measurement tools. Subgroup analysis was not conducted when fewer than three studies were included in the subgroup. A meta-regression, based on these factors, was performed to analyze the potential sources of heterogeneity. Sensitivity analysis was also performed by leave-one-out method and excluding studies with detection rates of IC deficits below 20% and above 90% to test the robustness of the study findings.

Moreover, we performed a narrative synthesis using data extracted from included studies to summarize tools used for IC measurement and illustrate the associated factors of IC deficits. We classified the associated factors into four aspects: socio-demographic factors, lifestyle factors, disease-related issues or subjective health conditions, and function-related conditions.

## Results

### Search results

**Fig. 1:**
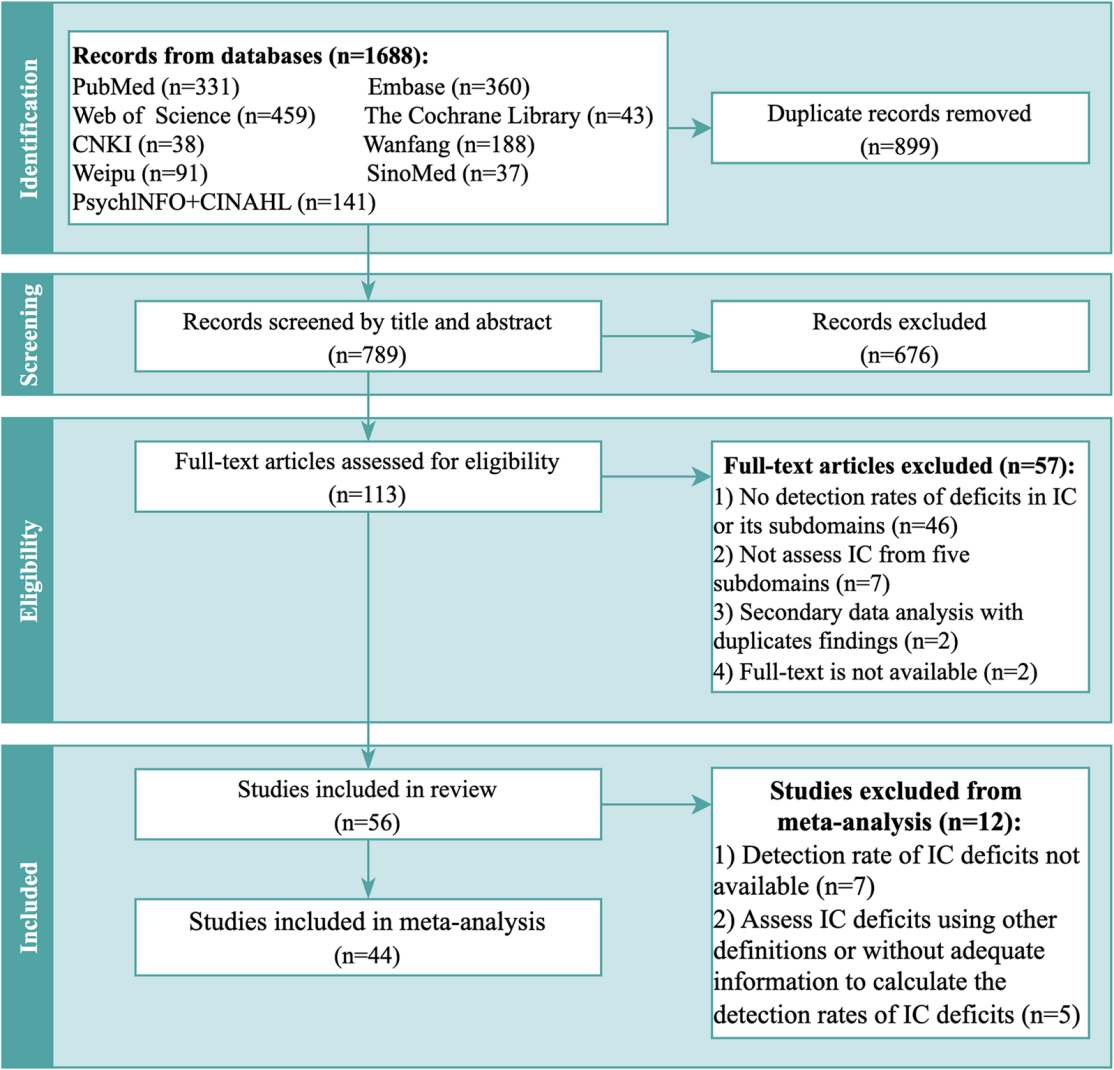
PRISMA flow diagram. Abbreviations: CNKI: China National Knowledge Infrastructure; SinoMed: Chinese Biomedical Literature Database; CINAHL: Cumulative Index to Nursing and Allied Health Literature; IC: intrinsic capacity

We identified 1,688 records from ten databases, and 789 records underwent screening process. After screening of title and abstracts, 113 studies were reviewed with full text and 56 studies were included in this review (Fig. 1). Of the 56 studies, 44 studies with information on the detection rates of IC deficits were included in the meta-analysis.

### Characteristics of included studies

A total of 56 studies from 13 countries were included (Table 1). Majority (73.2%) were from LMICs, such as China (*n* = 39), India (*n* = 3), Mexico (*n* = 2), and Brazil (*n* = 1). About 85.7% were from countries in an aged (73.2%) or hyper-aged society (12.5%), such as France (*n* = 4), Japan (*n* = 2), and Singapore (*n* = 2). Most studies were cross-sectional studies (73.2%), with 32.1% based on secondary data analysis. Community settings (55.4%) were the most common, followed by hospital settings (33.9%) and primary care facilities (10.7%).

**Table 1:** Summary of characteristics of included studies

Study characteristics	Number of studies (n, %)	
	Included in the review (*N* = 56)	Included in the meta-analysis (*N* = 44)
Economic status of countries		
HICs	15 (26.8)	12 (27.3)
LMICs	41 (73.2)	32 (72.7)
Continent		
Asia	46 (82.1)	38 (86.4)
North America	2 (3.6)	2 (4.5)
Central and South America	3 (5.4)	1 (2.3)
Europe	5 (8.9)	3 (6.8)
Stage of ageing society of countries		
Ageing society	8 (14.3)	4 (9.1)
Aged society	41 (73.2)	35 (79.6)
Hyper-aged society	7 (12.5)	5 (11.4)
Study design		
Cross-sectional study	41 (73.2)	31 (70.4)
Cohort study	14 (25.0)	12 (27.3)
Baseline survey of trial	1 (1.8)	1 (2.3)
Setting of data collection		
Hospital	19 (33.9)	19 (43.2)
Primary care facility	6 (10.7)	4 (9.1)
Community	31 (55.4)	21 (47.7)
Sample size		
< 1 000	38 (67.9)	32 (72.7)
1000 ~ 10,000	11 (19.6)	8 (18.2)
> 10,000	7 (12.5)	4 (9.1)
Secondary data analysis		
Yes	18 (32.1)	14 (31.8)
No	38 (67.9)	30 (68.2)
Intrinsic capacity measurement tool		
ICOPE step1	14 (25.0)	11 (25.0)
ICOPE step2	15 (26.8)	14 (31.8)
Others	27 (48.2)	19 (43.2)

*Abbreviations*: *HICs* high-income countries, *LIMCs* low- and middle- income countries, *ICOPE* Integrated Care of Older People

The 56 studies corresponded to 182,388 participants, averaging 74.2 years of age. The mean age of the participants ranged from 67.8 to 84.7 years. Sample size varied from 100 to 37,993, with 67.9% comprising studies with fewer than 1,000 participants. About eight studies only recruited individuals with health conditions, such as hypertension, acute coronary syndrome, a history of falls within the past 12 months, or limitations in activities [27–34] (Detailed characteristics were summarized in Table 2).

**Table 2:** Detection rates of deficits in intrinsic capacity and its subdomains in the 56 included studies

Author, year	Participants	Sample size	Minimum recruitment age	Mean age	Detection rate of intrinsic capacity deficits (%)	Detection rates of deficits in subdomains of intrinsic capacity (%)					
						Cognition	Locomotion	Psychology	Vitality	Vision	Hearing
Chang et al., 2023 [35]	Community-dwelling older adults	1268	60 +	NA	34.4	NA	NA	NA	NA	NA	NA
Chen et al., 2023 [36]	Community-dwelling older adults	810	50 +	68.5	94.7	56.0	48.0	25.3	7.4	61.4	13.2
Cheng et al., 2021 [27]	Older adults aged ≥ 65 years, combined with hypertension, diabetes, dyslipidemia or ≥ 75 years	457	65 +	73.0	17.1	5.5	2.8	2.0	2.2	7.0	1.8
García-Chanes et al., 2022 [37]	Community-dwelling older adults	590	65 +	76.6	43.0	50.6	49.9	47.3	35.1	48.3	1.2
Gaussens et al., 2023 [38]	Community-dwelling older adults	14,572	60 +	76.7	NA	60.7	37.0	39.6	20.4	75.0	63.2
González-Bautista et al., 2021 [28]	Community-dwelling older adults	759	70 +	75.2	89.3	52.2	20.2	39.0	6.6	18.1	56.2
Gonzalez-Bautista et al., 2023 [39]	Community-dwelling older adults	14,923	65 +	74.5	NA	22.4	32.8	23.7	12.8	30.4	15.9
Gutiérrez-Robledo et al., 2021 [40]	Community-dwelling older adults	12,459	50 +	71.2	87.8	37.2	47.6	43.1	27.5	44.8	32.1
Huang et al., 2022 [30]	Hypertensive patients in geriatric department	206	NA	72.5	64.1	NA	NA	NA	NA	NA	NA
Jia et al., 2023 [41]	Community-dwelling older adults	808	60 +	67.8	75.1	11.4	36.5	17.0	19.4	44.6	
Jiang et al., 2023a [42]	Community-dwelling older adults	1042	60 +	72.8	72.9	21.0	32.2	5.3	11.6	51.0	
Jiang et al., 2023b [43]	Community-dwelling older adults	485	60 +	69.9	NA	25.4	4.7	16.7	10.9	64.3	2.7
Jiang et al., 2023c [44]	Community-dwelling older adults	968	65 +	71.0	72.7	19.7	31.4	5.3	11.1	52.4	
Leung et al., 2022 [13]	Community-dwelling older adults	304	60 +	76.7	72.7	24.3	37.8	35.2	18.1	8.9	14.5
Li et al., 2021 [29]	Patients with ASC in Danzhou People's Hospital	221	60 +	73.7	68.3	NA	NA	NA	NA	NA	NA
Lin et al., 2022 [45]	Community-dwelling older adults	1927	60 +	72.0	33.6^a^	NA	NA	NA	NA	69.3	
Lin et al., 2023 [46]	Community-dwelling older adults	1972	60 +	71.0	34.1^a^	NA	NA	NA	NA	NA	NA
Liu et al., 2021a [47]	Community-dwelling older adults	230	75 +	84.0	67.9	16.3	58.2	14.8	14.3	8.7	
Liu et al., 2021b [48]	Community-dwelling older adults	212	75 +	83.8	51.4	18.9^d^, 49.1^e^	46.7	14.2^f^, 29.7^ g^	6.6^ h^, 6.6^i^	19.3	18.9
Liu et al., 2022 [49]	Older patients in Geriatric Hospital Affiliated to Nanjing Medical University	356	70 +	84.7	70.8	NA	NA	NA	NA	NA	NA
Lu et al., 2023 [50]	Community-dwelling older adults	228	75 +	84.0	73.2	17.1	60.1	16.2	17.1	19.7	20.2
Ma et al., 2020 [11]	Older patients in geriatric department of Xuanwu Hospital	376	50 +	68.7	69.1	46.8	25.3	12.0	16.2	11.7	15.4
Ma et al., 2021a [51]	No acute disease relatively healthy older adults in hospital	283	60 +	77.4	75.3	51.6	31.4	13.1	18.4	13.8	18.4
Ma et al., 2021b [52]	Older adults in hospital	130	60 +	73.1	19.2	NA	NA	NA	NA	NA	NA
Ma et al., 2021c [53]	Community-dwelling older adults	5823	60 +	NA	39.9	11.1	17.8	12.2	12.6	14.2	
Ma et al., 2023 [54]	Community-dwelling older adults	868	65 +	72.4	72.0	14.0	28.7	4.2	10.9	49.3	
Mathur et al., 2022 [55]	The rural older adults	451	60 +	68.4	NA	31.5	52.1	19.3	17.5^ h^, 33.7^i^	49.4	68.3
Meng et al., 2022 [56]	Community-dwelling older adults	37,993	65 +	73.2	40.6	20.3	11.2	7.6	4.6	17.6	10.8
Merchant et al., 2022 [31]	Community-dwelling older adults	154	60 +	74.6	NA	59.9	57.2	27.9	29.9	78.5	21.6
Muneera et al., 2023 [57]	Community-dwelling older adults	24,136	60 +	NA	75.4^b^	NA	NA	NA	NA	NA	NA
Nagae et al., 2023 [58]	Older inpatients in geriatric department	296	65 +	84.7	95.6	73.6	55.7	51.7	74.7	14.2	22.0
Pagès et al., 2022 [32]	Non-demented community-dwelling older adults	693	70 +	75.2	89.0	52.0	30.0	38.0	7.0	18.0	56.0
Plácido et al., 2023 [59]	Community-dwelling older adults	9070	50 +	NA	25.2^a^	27.8	33.9	28.9	25.6	73.2	
Prince et al., 2021 [60]	Community-dwelling older adults	17,031	65 +	NA	70.4	26.5	28.8	25.9	15.5	28.7	15.2
Rarajam Rao et al., 2023 [61]	Community-dwelling older adults	1000	60 +	66.5	84.3	10.6	59.3	3.8	3.7	44.1	19.3
Rojano et al., 2023 [62]	Community-dwelling older adults	207	70 +	76.7	70.0	31.0	28.0	24.0	11.0	34.0	32.0
Saiyare et al., 2023 [63]	Community-dwelling older adults	1072	60 +	72.0	73.4	47.3	39.6	12.0	18.8	4.8	8.6
Shi et al., 2023 [64]	Older patients in the geriatrics department	370	60 +	81.9	87.6	25.4	67.3	12.2	23.2	58.1	35.9
Sun et al., 2022 [65]	Older inpatients in geriatric department of Beijing Hospital	264	60 +	81.0	94.3	NA	NA	NA	NA	NA	NA
Tang et al., 2023 [34]	Older adults diagnosed with T2DM	2482	65 +	72.4	38.6	NA	NA	NA	NA	NA	NA
Tavassoli et al., 2022 [10]	Community-dwelling older adults	958	60 +	80.4	NA	65.0	86.4	44.8	77.2	39	41.3
Tay et al., 2023 [66]	Community-dwelling older adults	809	55 +	67.6	73.7	35.6	20.8	20.9	30.4	20.8	16.7
Wang et al., 2022 [67]	Community-dwelling older adults	236	60 +	72.2	44.1^c^	27.5	7.2	19.1	13.6	94.1	
Wu et al., 2022 [68]	Older inpatients in geriatric department of Beijing Hospital	909	60 +	76.6	98.0	30.7	91.1	57.3	29.2	61.4	
Yang et al., 2023 [69]	Older inpatients in geriatric department of Zhejiang Hospital	311	60 +	78.3	90.7	28.6	22.5^j^, 55.9^ k^	11.9	26.7^ l^, 30.2^ m^	62.1	43.4
You et al., 2023 [70]	Older inpatients in geriatrics department	100	60 +	71.4	93.0	NA	NA	NA	NA	NA	NA
Yu et al., 2021 [71]	Community-dwelling older adults	756	60 +	69.3	NA	4.5	22.1	13.9	6.9	26.7	13.5
Yu et al., 2022 [72]	Community-dwelling older adults	10,007	60 +	75.7	85.3	71.3	45.8	16.9	6.1	22.8	19.1
Zhang et al., 2020a [73]	Older inpatients in geriatrics department of Beijing Hospital	196	60 +	80.1	93.4	NA	NA	NA	NA	NA	NA
Zhang et al., 2020b [74]	Older inpatients in geriatrics department of Beijing Hospital	125	60 +	81.8	92.0	36.0	68.8	29.6	40.8	46.4	
Zhang et al., 2023a [75]	Older patients in geriatrics department of Beijing Hospital	267	60 +	81.0	95.5	37.1	73.4	18.7	51.3	65.5	
Zhang et al., 2023b [33]	Older patients with chronic noncommunicable diseases from the First Affiliated Hospital of Zhengzhou University	322	75 +	81.0	59.0	NA	NA	NA	NA	NA	NA
Zhang et al., 2023c [76]	Community-dwelling older adults	1640	60 +	75.4	81.5	NA	NA	NA	NA	NA	NA
Zhao et al., 2021 [77]	Community-dwelling older adults	7298	65 +	74.2	64.5	18.4	11.1	11.8	34.9	32.8	
Zhao et al., 2023 [78]	Community-dwelling older adults	577	60 +	72.5	87.0	NA	NA	NA	NA	NA	NA
Zhu et al., 2023 [79]	Community-dwelling older adults	381	60 +	82.0	76.9	22.6	63.5	11.3	18.9	27.3	

*Abbreviations*: *NA* not available

^a^Define intrinsic capacity deficits as declines in two or more subdomains

^b^Define intrinsic capacity deficits as below 9 scores in intrinsic capacity

^c^Define intrinsic capacity deficits as declines in three or more subdomains

^d^Detection rate of having problem in time orientation

^e^Detection rate of having problem in memory

^f^Detection rate of feeling down, depressed or hopeless

^g^Detection rate of having little interest

^h^Detection rate of weight loss

^i^Detection rate of appetite loss

^j^Detection rate of having problem in balance

^k^Detection rate of having problem in gait speed

^l^Detection rate of having problem in grip strength

^m^Detection rate of having problem in nutrition

### Intrinsic capacity measurement tools in included studies

As illustrated in Supplementary Table 1, a consensus on the measurement tools for individual subdomains of IC has not been established, and various studies used diverse measurement tools to assess each subdomain of IC. For instance, the Mini-Mental State Examination (MMSE) [80] was the most common scale used to measure cognition, while the Montreal Cognitive Assessment (MoCA) [81] and other scales were also used. Studies commonly applied the Short Physical Performance Battery (SPPB) test [82] for the assessment of locomotion. The Mini Nutritional Assessment (MNA) [83] and its short form (MNA-SF) [84] were the most commonly used scales for assessing vitality. Psychological assessments typically employed the Geriatric Depression Scale (GDS) [85] or Patient Health Questionnaire-9 (PHQ-9) [86]. Sensory assessments relied mainly on self-reported status of problems.

### Detection rates of intrinsic capacity deficits

As displayed in Table 2, the detection rates of IC deficits among the 56 included studies varied widely, ranging from 17.1% to 98.0%. The detection rate of deficits in cognition, locomotion, psychology, vitality, and sensory ranged from 4.5% to 73.6%, 2.8% to 91.1%, 2.0% to 57.3%, 2.2% to 77.2%, and 8.7% to 94.1%, respectively.

**Fig. 2:**
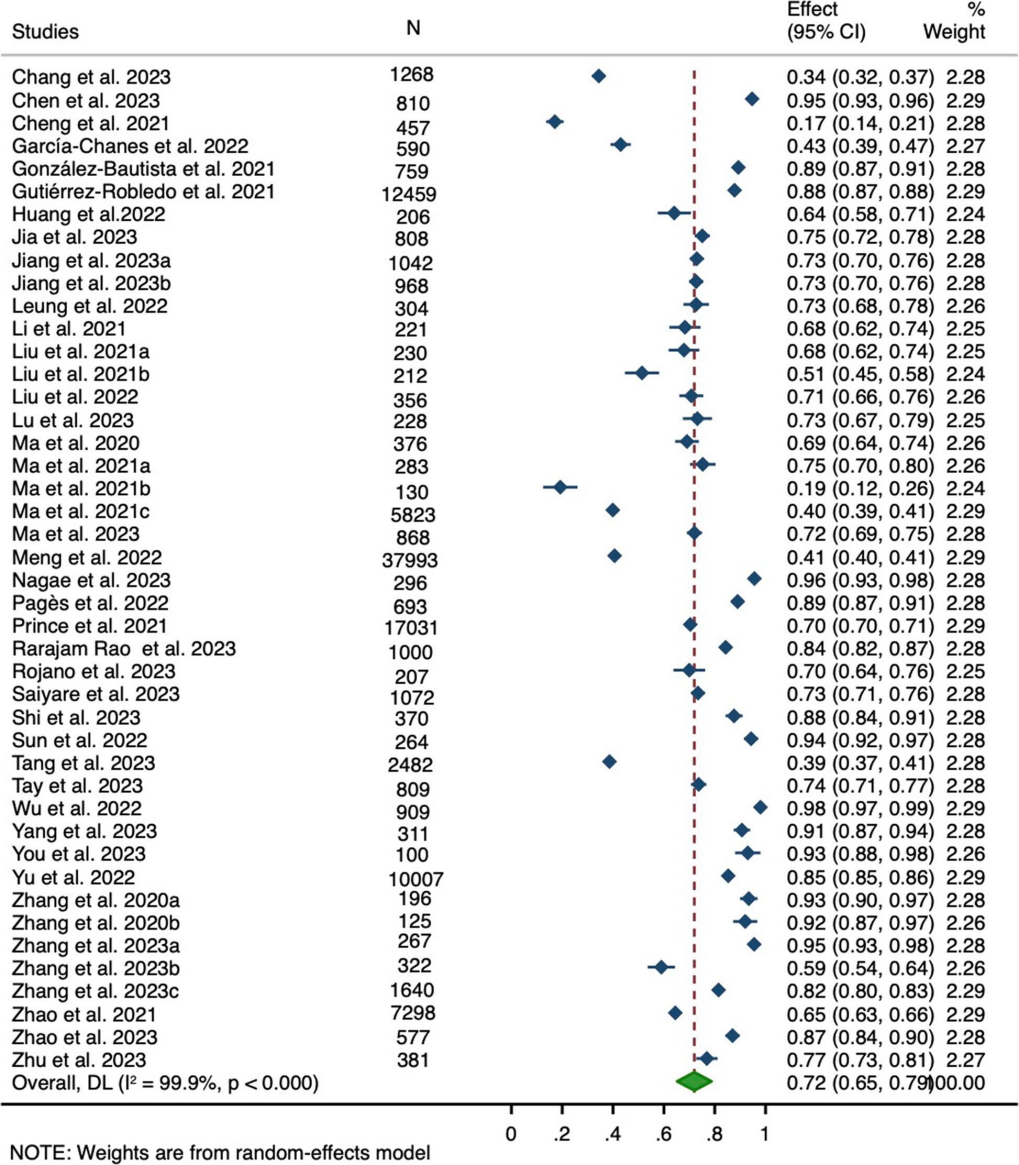
Forest plot of the detection rate of intrinsic capacity deficits among 44 studies that reported the detection rates of intrinsic capacity deficits. Abbreviations: CI: confidence interval

The 44 studies with available detection rates of IC deficits pooled a total of 112,748 participants. The overall pooled detection rate of IC deficits was 72.0% (95% CI: 65.2%-78.8%) but with high heterogeneity (*I*^2^ = 99.9%, *P* < 0.001) (Fig. 2). Across subdomains of IC, the pooled detection rate of deficits was highest in sensory (49.3%, 95% CI: 34.2%-64.4%; [Vision: 33.6%, 95% CI:25.8%-41.3%; Hearing: 24.8%, 95% CI: 19.1%-30.6%]), followed by locomotion (40.0%, 95% CI: 34.1%-45.8%), cognition (33.1%, 95% CI: 27.5%-38.7%), psychology (21.9%, 95% CI: 17.9%-25.9%) and vitality (20.7%, 95% CI: 17.4%-24.0%).

### Subgroup analyses and meta-regression

The findings of a series of subgroup analyses on the pooled detection rate of IC deficits were reported in Table 3. The pooled detection rate of IC deficits among studies conducted in LMICs (74.0%, 95% CI: 68.2%-79.8%) was slightly higher than that in HICs (66.8%, 95% CI: 50.2%-83.3%). For countries with different stages of ageing society, the detection rate of IC deficits was highest in hyper-aged societies at 85.0% (95% CI: 78.0%-91.9%), followed by ageing societies at 71.5% (95% CI: 59.0%-84.1%) and aged societies (70.2%, 95% CI: 61.7%-78.8%).

**Table 3:** Subgroup analyses by country, setting of data collection and measurement tools of intrinsic capacity

Variables	Number of studies	Pooled detection rate	95% CI	*I*^2^, %
By countries’ characteristics				
Economic status of countries				
HICs	12	66.8%	50.2%-83.3%	99.8%
LMICs	32	74.0%	68.2%-79.8%	99.4%
Stage of ageing society of countries				
Ageing society	4	71.5%	59.0%-84.1%	99.8%
Aged society	35	70.2%	61.7%-78.8%	99.9%
Hyper-aged society	5	85.0%	78.0%-91.9%	97.0%
By data collection methods				
Setting of data collection				
Hospital	19	73.7%	61.9%-85.4%	99.7%
Primary care facility	4	80.6%	71.5%-89.7%	97.6%
Community	21	68.9%	59.3%-78.4%	99.9%
Intrinsic capacity measurement tool				
ICOPE step1	11	62.3%	45.0%-79.6%	99.7%
ICOPE step2	14	79.1%	73.2%-84.9%	97.4%
Others	19	72.4%	61.9%-82.9%	99.9%

*Abbreviations*: *IC* intrinsic capacity, *CI* confidence interval, *HICs* high-income countries, *LMICs* low- and middle-income countries, *ICOPE* Integrated Care for Older People

The pooled detection rate of IC deficits also varied across different data collection settings and measurement tools. The pooled detection rate of IC deficits was 80.6% (95% CI: 71.5%-89.7%) among older adults recruited from primary care facilities, which was relatively higher than those from hospitals (73.7%, 95% CI: 61.9%-85.4%) and communities (68.9%, 95% CI: 59.3%-78.4%). Among 25 studies that used ICOPE tools, the pooled detection rate was 71.6% (95% CI: 62.6%-80.7%) (Supplementary Fig. 1), with 62.3% (95% CI: 45.0%-79.6%) and 79.1% (95% CI: 73.2%-84.9%) for 11 and 14 studies that used ICOPE step 1 and step 2 assessment tools respectively. Across 19 studies that used other IC measurement tools, the pooled rate was 72.4% (95% CI: 61.9%-82.9%).

The result of meta-regression revealed that the stage of ageing society of countries was associated with the heterogeneity of the IC deficits, which could explain 7.75% of heterogeneity. (Supplementary Table 2).

### Methodological quality and publication bias

**Fig. 3:**
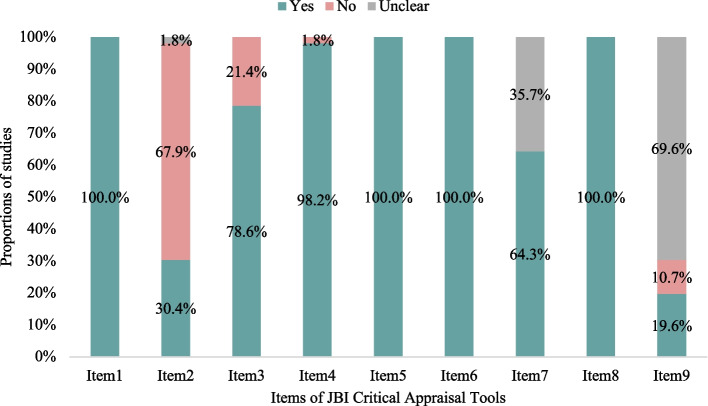
Assessment results of each item of JBI (Joanna Briggs Institute) Critical Appraisal Tool. Item1: Was the sample frame appropriate to address the target population? Item2: Were study participants sampled in an appropriate way? Item3: Was the sample size adequate? Item4: Were the study subjects and the setting described in detail? Item5: Was the data analysis conducted with sufficient coverage of the identified sample? Item6: Were valid methods used for the identification of the condition? Item7: Was the condition measured in a standard, reliable way for all participants? Item8: Was there appropriate statistical analysis? Item9: Was the response rate adequate, and if not, was the low response rate managed appropriately?

As shown in Fig. 3, the overall scores of the 56 included studies ranged from five to nine, with 55.4% of studies reaching a high level of quality (Supplementary Table 3 shows the rating details for each study). The significant methodological weaknesses included using a convenient sampling approach (37, 66.1%) and the absence of a response rate (39, 69.6%) in the original studies.

The funnel plot showed a potential asymmetry in 44 studies included in the meta-analysis, while the Egger’s test results showed the absence of publication bias for 44 studies reporting the detection rate of IC deficits (t = 0.74, *P* = 0.462) (Supplementary Fig. 2), as well as in most subgroup analyses, except for those conducted in a hyper-aged society (t = -4.04, *P* = 0.027).

### Sensitivity analysis

The sensitivity analysis showed the robustness of the study findings. No discernible change was observed by employing the leave-one-out method to scrutinize potential influence caused by individual study. The pooled detection rate was only slightly lower (69.2%, 95% CI: 61.7%-76.6%) after removing studies with detection rates of IC deficits below 20% and above 90% (Supplementary Fig. 3).

### Key associated factors of intrinsic capacity

**Fig. 4:**
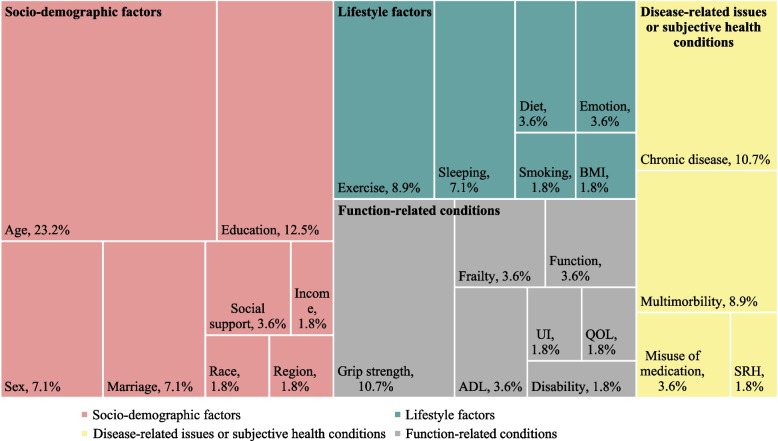
Key associated factors with intrinsic capacity and percentage of corresponding studies. Abbreviations: BMI: Body Mass Index; SRH: Self-reported health; ADL: Activities of daily living; UI: Urinary incontinence; QOL: Quality of life

Figure 4 illustrated the associated factors or outcomes with IC deficits examined in the 56 studies. A large proportion of studies focused on the influence of socio-demographic factors on IC, including age, marrital status, education level, etc., while some lifestyle factors, such as exercise and sleep behaviors, were also examined. Studies also illustrated the potential outcomes of IC deficits in both disease-related conditions, such as chronic diseases and multimorbidity, and function-related conditions, such as frailty, disability, and activities of daily living.

## Discussion

This systematic review synthesized the evidence regarding the detection rate of IC deficits among older adults on a global scale. Our review extended the existing review by including 56 studies conducted in 13 countries, quantifying the variation of IC deficits by study population and methodologies, and illustrating factors that associated with IC deficits. We observed a substantial pooled detection rate of IC deficits (72.0%) among older adults, with more issues in sensory, locomotion and cognition across all five subdomains. The detection rates of IC deficits varied across studies conducted in different countries and employing different data collection methods. The findings of this study illustrated the importance of assessing IC among older adults as a means of early detection and intervention to maintain functional ability among older adults.

Our study illustrated a high heterogeneity in IC deficits across countries and population groups. Consistent with previous studies that indicated socioeconomic status may influence IC among older adults [87], our study observed a relatively higher pooled detection rate of IC deficits among older adults in LMICs compared to those in HICs. We also observed a relatively higher prevalence of IC deficits in countries that classified into hyper-aged societies. Although many factors may influence the disparities in observed detection rates of IC across countries and settings, such findings are worth special attention. The higher prevalence of IC deficits in LMICs and hyper-aged societies highlights that the magnitude of the problem could be different across countries and LMICs may bear more burden. Many of the LMICs are experiencing demographic transition and population ageing, while their healthcare and social care system have not been prepared enough for such transition and increasing needs. Barriers may exist in multiple levels, including unavailability and inaccessibility of geriatric care, insufficient health workforce, lack of structural healthcare and social supports, etc. [88, 89]. These findings emphasize the critical and pressing needs of IC assessment and intervention among older population particularly in LMICs and countries undergoing rapid population ageing.

Our study also revealed the large variation in assessment tools and methods employed in existing studies. Consistent with existing reviews [14, 15], our study also highlights the issue of the absence of a standardized tool for assessing IC and its subdomains. It is worth noting that we found an increasing number of studies applied ICOPE assessment tools in IC assessment [11, 35, 48, 62]. These studies illustrated a tendency to use ICOPE step 1 tool in community settings to perform screening of IC [13, 35, 36, 48], while step 2 tool with detailed scales in subdomain assessment were more likely to be used in hospital settings or in primary healthcare facilities, as well as in cohort studies that aimed to have an intensive assessment of IC [29, 30, 64, 65, 73–75]. This tendency may partially explain the observed higher pooled detection rates of IC deficits in studies that used ICOPE step 2 tools or other valid tools than in studies that used ICOPE step 1 tool. Notably, the rate of IC deficit remained significant in studies that conducted in general communities, which further underscores the significance of IC deficits among general older adults and the importance of performing early detection of IC.

Our review identified several important research gaps in the evidence, which shed light for future research. Firstly, despite the increasing number of studies, the majority originated from a limited set of 13 countries, with China, France and India accounted for more than 80% of the identified studies. Besides, many existing studies were small in size and confined to single study settings, limiting the generalizability of findings [10, 47, 55]. Thus, studies are needed to assess IC in various settings on a larger scale to enhance the overall understanding of IC deficits across diverse population groups. Secondly, only five studies assessed IC in adults under 60 years old [11, 36, 40, 59, 66]. Given evidence suggesting early onset of IC deficits [7], future research could pay attention to younger older populations with repeated measures to track IC trajectories during middle-age. Thirdly, we identified a series of socio-demographic and health-related factors with potential association with IC. However, only four studies in our review were cohort studies with repeated assessments of IC and key factors [10, 39, 41, 47]. Future research could further explore the causal relationship between risk factors and IC deficits, as well as the long-term health outcomes related with IC deficits.

Furthermore, our study also provided some insights for implementing assessment and early intervention of IC in routine practice. The increasing and widely use of WHO ICOPE tools across studies and various settings suggest a general feasibility and the great potential of scaling up ICOPE tools in various settings [7]. The WHO ICOPE step 1 tool, a simple and time-efficient tool, could be used in community settings for screening of general population. The ICOPE step 2 tools contain further assessment by using valid scales for different subdomains, are more applicable to be used by health professionals in the healthcare settings. Future studies are needed to examine how ICOPE tools could be better integrated into the service delivery in both community and hospital settings, along with relevant trainings and capacity building provided to community-based workers and healthcare professionals. Besides, the use of modern information and communication technologies, such as wearable devices or self-assessment applications should also be explored, as some studies have indicated their great potential [10, 90]. As many of the included studies were designed for observational purpose only, fostering partnerships among healthcare providers, community-based practitioners and researchers is also crucial to share the resources and best practice, so as to promote the implementation of IC assessment and interventions in different contexts.

Our study had several strengths and real-world implications. Our review captured the latest studies with an extensive search across ten major databases encompassing both Chinese and English literature and provided a global mapping of existing evidence. This review added to the evidence base by not only showing the diversity in measurement but also quantifying the detection rate of IC deficits for different types of studies that used various measurement tools and approaches. In addition, our study performed meta-analyses of detection rates for both IC and its subdomains, which allowed us to identify susceptible subdomains. These findings could be valuable for designing more precise measures for early prevention of IC deficits.

However, our systematic review also bears some limitations. Firstly, the included studies in our review exhibited substantial heterogeneity, which might reduce the robustness of our findings. However, we conducted subgroup analysis and meta-regression to explore potential sources of heterogeneity. Secondly, we chose the detection rate of IC deficits as a binary outcome to quantitively synthesize studies that used different methods in IC scoring. This analytical method may weaken the differences in the degree of IC deficits across individuals, but allowed for a comparison of broader studies with different measures. Lastly, for 14 cohort studies, we only extracted data from the baseline survey in our analysis. Future research could further examine the trajectory of IC over time [7].

## Conclusion

In conclusion, our review provided a global snapshot of studies that reported the status of IC deficits across countries, and demonstrated a high prevalence with great variation in IC deficits across countries and by methods. Moving forward, implementing IC assessment could be crucial for many countries, especially LMICs and countries that experiencing rapid population ageing. To better implement early screening and assessment of IC, more efforts are needed in scaling-up WHO ICOPE tools to support comparison across studies, providing trainings on IC screening and assessment to both healthcare professionals and community workers, and improving the awareness and joint efforts in building an integrated care for healthy ageing.

### Supplementary Information

Additional file 1. Search strategy.

Additional file 2: Supplementary Fig. 1. Forest plot of the detection rate of intrinsic capacity deficits among 25 studies used ICOPE tools. Supplementary Fig. 2. (A) Funnel plot of 44 studies that reported the detection rates of intrinsic capacity deficits; (B) Funnel plot of 25 studies that used ICOPE tools to assess intrinsic capacity. Supplementary Fig. 3. Sensitivity analysis by removing studies with detection rates of intrinsic capacity deficits below 20% and above 90%. Supplementary Table 1. Measurement tools and methods used for intrinsic capacity subdomains among included studies. Supplementary Table 2. Meta-regression analyses result. Supplementary Table 3. Methodological quality of the 56 included studies.

## Data Availability

This study was based on the data extracted from previously published studies; most of the data and study materials of which are available in the public domain. For further discussion, please contact the corresponding author.

## Abbreviations

IC: Intrinsic capacity
ICOPE: Integrated Care for Older People
CNKI: China National Knowledge Infrastructure
SinoMed: Chinese Biomedical Literature Database
CINAHL: Cumulative Index to Nursing and Allied Health Literature
HICs: High-income countries
LIMCs: Low- and middle- income countries
CI: Confidence interval
NA: Not available
BMI: Body Mass Index
SRH: Self-reported health
ADL: Activities of daily living
UI: Urinary incontinence
QOL: Quality of life
