# Supplementary material for: The assessment and detection rate of intrinsic capacity deficits among older adults: a systematic review and meta-analysis
Source: BMC Geriatr. 2024 Jun 3;24:485. doi: 10.1186/s12877-024-05088-w (PMC11149255; doi:10.1186/s12877-024-05088-w)
Supplement: Supplementary file 1 — Additional file 1. Search strategy. [file 12877_2024_5088_MOESM1_ESM.pdf]

Supplementary file 1. Search strategy

No.	Query
PubMed	
#1	"elderly"[Title/Abstract] OR "Aged"[Title/Abstract] OR "aging"[Title/Abstract] OR "ageing"[Title/Abstract] OR "old"[Title/Abstract] OR "Older"[Title/Abstract] OR "middle age*"[Title/Abstract] OR "senior*"[Title/Abstract] OR "geriatric*"[Title/Abstract] OR "Aged"[MeSH Terms]
#2	"intrinsic capacit*"[Title/Abstract]
#3	"locomotion"[Title/Abstract] OR "locomotor capacity"[Title/Abstract]
#4	"psychology"[Title/Abstract] OR "psychological capacity"[Title/Abstract]
#5	"cognition"[Title/Abstract] OR "cognitive capacity"[Title/Abstract]
#6	"vitality"[Title/Abstract]
#7	"sensory"[Title/Abstract] OR "visual capacity"[Title/Abstract] OR "hearing capacity"[Title/Abstract]
#8	#3 AND #4 AND #5 AND #6 AND #7
#9	"integrated care for older people"[Title/Abstract] OR "ICOPE"[Title/Abstract]
#10	#2 OR #8 OR #9
#11	#1 AND #10
#12	#1 AND #10 AND (2015:2023[pdat])
Embase	
#1	elderly:ab,ti OR aging:ab,ti OR ageing:ab,ti OR old:ab,ti OR older:ab,ti OR 'middle age':ab,ti OR senior:ab,ti OR geriatric:ab,ti OR aged:ab,ti
#2	'intrinsic capacit*':ab,ti
#3	'locomotor capacity':ab,ti OR locomotion:ab,ti
#4	'psychological capacity':ab,ti OR psychology:ab,ti
#5	'cognitive capacity':ab,ti OR cognition:ab,ti
#6	vitality:ab,ti
#7	'visual capacity':ab,ti OR sensory:ab,ti OR 'hearing capacity':ab,ti
#8	#3 AND #4 AND #5 AND #6 AND #7
#9	'integrated care for older people':ab,ti OR icope:ab,ti
#10	#2 OR #8 OR #9
#11	#1 AND #10
#12	#1 AND #10 AND [2015-2023]/py
Web of Science	
#1	TS=(elderly or aging or ageing or old or older or "middle age" or senior or geriatric or aged)
#2	TS="intrinsic capacit*"
#3	TS=("locomotor capacity" or locomotion)
#4	TS=("psychological capacity" or psychology)
#5	TS=("cognitive capacity" or cognition)
#6	TS=vitality
#7	TS=("visual capacity" or "hearing capacity" or sensory)
#8	#3 AND #4 AND #5 AND #6 AND #7

#9	TS=("integrated care for older people" or ICOPE)
#10	#2 OR #8 OR #9
#11	#10 AND #1
#12	#10 AND #1 AND PY=(2015-2023)
The Cochrane Library	
#1	(elderly or aging or ageing or old or older or "middle age" or senior or geriatric or aged):ti,ab,kw
#2	("intrinsic capacity"):ti,ab,kw
#3	("locomotor capacity" or locomotion):ti,ab,kw
#4	("psychological capacity" or psychology):ti,ab,kw
#5	("cognitive capacity" or cognition):ti,ab,kw
#6	vitality:ti,ab,kw
#7	("visual capacity" or "hearing capacity" or sensory):ti,ab,kw
#8	#3 AND #4 AND #5 AND #6 AND #7
#9	"integrated care for older people" or ICOPE):ti,ab,kw
#10	#2 OR #8 OR #9
#11	#10 AND #1
#12	#10 AND #1 AND PY=(2015-2023)
PsycINFO and CIANHL	
#1	elderly or aging or ageing or old or older or "middle age" or senior or geriatric or aged AB
#2	"intrinsic capacit*" AB
#3	"locomotor capacity" or locomotion AB
#4	"psychological capacity" or psychology AB
#5	"cognitive capacity" or cognition AB
#6	vitality AB
#7	"visual capacity" or "hearing capacity" or sensory AB
#8	#3 AND #4 AND #5 AND #6 AND #7
#9	"integrated care for older people" or ICOPE AB
#10	#2 OR #8 OR #9
#11	#10 AND #1
#12	#10 AND #1 AND PY=(2015-2023)
China Knowledge Resource Integrated Database	
（主题：老年人 OR 老年）AND（主题：内在能力）；资源范围：总库；同义词扩展；时间范围：发表时间：2015 到 2023;更新时间：不限<br>(Topic: older adults OR ageing) AND (Topic: intrinsic capacity); Resource scope: general database; Synonym expansion; Time range: Published time: 2015 to 2023; Update time: unlimited.	
Wanfang Database	
主题:（老年人 OR 老年）AND 主题:（内在能力）；限制条件：期刊+主题词扩展+(2015 – 2023)<br>Topic: (older adults OR ageing) AND Topic: (intrinsic capacity); Limitations: Journals + Subject Heading Expansion + (2015 - 2023)	
Weipu Database	
R=（老年人 OR 老年）AND R=（内在能力）	

R = (older adults OR ageing) AND R = (intrinsic capacity)
SinoMed
（ "老年人"[摘要:智能] OR "老年"[摘要:智能]） AND "内在能力"[摘要:智能]<br>("older adults" [Abstract: Smart] OR "ageing" [Abstract: Smart]) AND "intrinsic capacity" [Abstract: Smart]
