# Supplementary material for: The assessment and detection rate of intrinsic capacity deficits among older adults: a systematic review and meta-analysis
Source: BMC Geriatr. 2024 Jun 3;24:485. doi: 10.1186/s12877-024-05088-w (PMC11149255; doi:10.1186/s12877-024-05088-w)
Supplement: Supplementary file 2 — Additional file 2: Supplementary Fig. 1. Forest plot of the detection rate of intrinsic capacity deficits among 25 studies used ICOPE tools. Supplementary Fig. 2. (A) Funnel plot of 44 studies that reported the detection rates of intrinsic capacity deficits; (B) Funnel plot of 25 studies that used ICOPE tools to assess intrinsic capacity. Supplementary Fig. 3. Sensitivity analysis by removing studies with detection rates of intrinsic capacity deficits below 20% and above 90%. Supplementary Table 1. Measurement tools and methods used for intrinsic capacity subdomains among included studies. Supplementary Table 2. Meta-regression analyses result. Supplementary Table 3. Methodological quality of the 56 included studies. [file 12877_2024_5088_MOESM2_ESM.pdf]

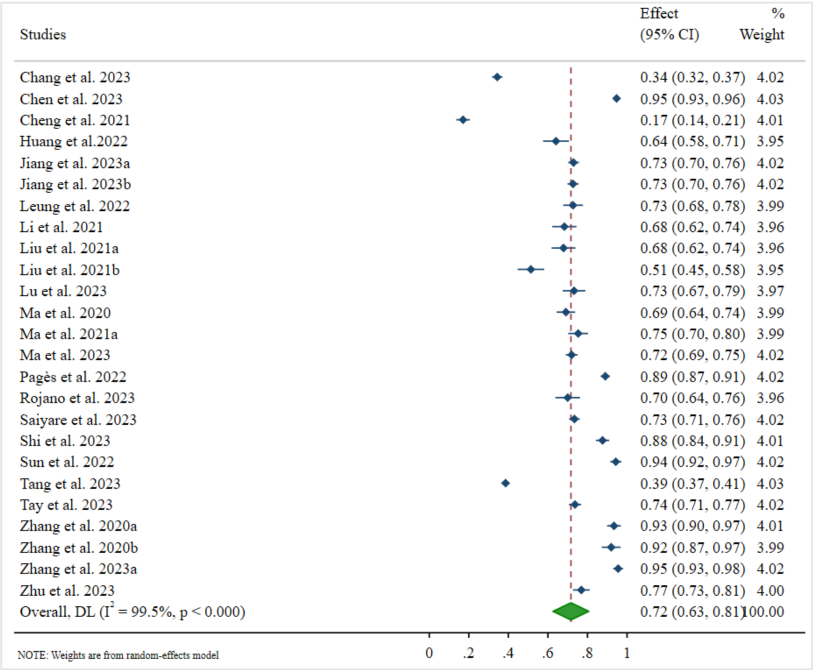

Supplementary Fig. 1. Forest plot of the detection rate of intrinsic capacity deficits among 25 studies used ICOPE tools.

Abbreviations: CI: confidence intervals; ICOPE: Integrated Care of Older People.

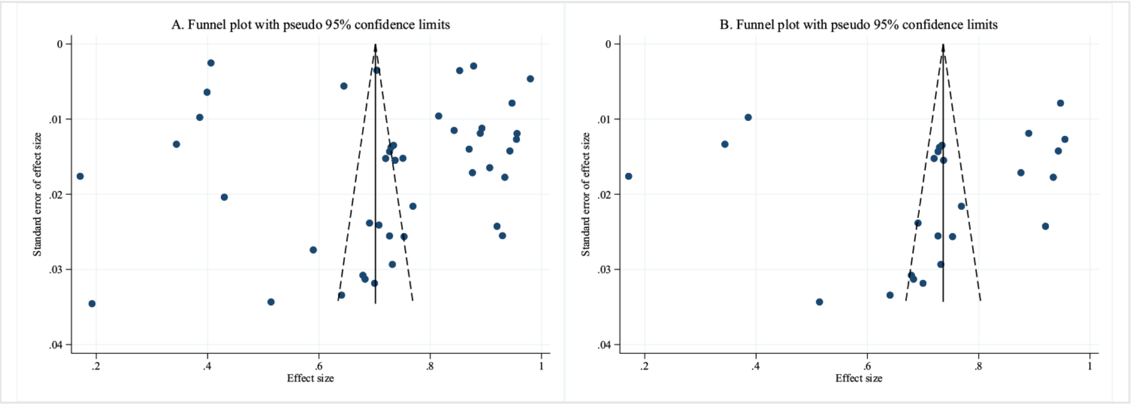

Supplementary Fig. 2. (A) Funnel plot of 44 studies that reported the detection rates of intrinsic capacity deficits; (B) Funnel plot of 25 studies that used ICOPE tools to assess intrinsic capacity.

Abbreviations: ICOPE: Integrated Care of Older People.

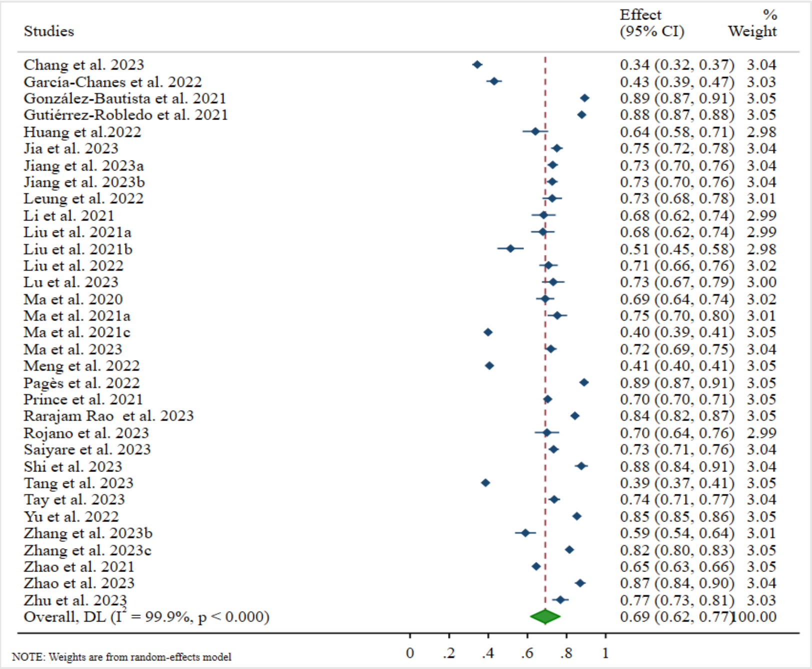

Supplementary Fig. 3. Sensitivity analysis by removing studies with detection rates of intrinsic capacity deficits below 20% and above 90%.

Abbreviations: CI: confidence intervals.

Supplementary Table 1 Measurement tools and methods used for intrinsic capacity subdomains among included studies.

Subdomains	ICOPE step1 screening tool [1-14]	ICOPE step2 assessment tool [15-30]	Others measurement tool
Cognition	Working memory, orientation, memory recall	Mini-Mental State Examination (MMSE)	Montreal Cognitive Assessment (MoCA)[31]<br>Mini-Cognitive Assessment Instrument (Mini-cog)[32-34]<br>Community screening instrument-dementia (CSI-D)[35, 36]<br>5-item Abbreviated Memory Inventory for Chinese (AMIC)[37]<br>Hachinski Ischemic Scale-Revised (HDS-R)[38]<br>Verbal Fluency Test[39]<br>Short Portable Mental Status Questionnaire (SPMSQ)
Locomotion	Chair rise test within 14s	Short Physical Performance Battery (SPPB)	Time up and go[2, 38, 40]<br>Gait speed[2, 33-36, 39]<br>Chair rest test within 12s[41]<br>Item 2 and item 3 from FRAIL scale[37]<br>Tinetti Performance-Oriented Mobility Assessment[42, 43]
Vitality	Weight loss, recent appetite loss	Mini Nutritional Assessment-Short Form (MNA-SF)	Mutritional Form For the Elderly (NUFFE)[44]<br>Loss of weight[35-37, 45, 46]<br>Body mass index (BMI)[45-47]<br>Skeletal muscle mass[25]<br>Grip strength[39, 42]<br>Biceps circumference[36]<br>Simplified Nutritional Appetite Questionnaire (SNAQ)[31]

Psychology	Feeling down/ depressed/ hopeless or little interest in doing things	Geriatric Depression Scale (GDS)	Patient Health Questionnaire-9 (PHQ-9)[32, 48]<br>Self-rating depression scale (SDS)[46]<br>UCLA Loneliness Scale, University of California at Los Angels (UCLA)[44]<br>European Depression Scale (EURO-D)[35, 36]<br>Center for Epidemiologic Studies Depression Scale (CES-D)<br>The anxiety dimension from the EQ-5D[25]
Sensory	Hearing loss, vision loss	Self-reporting problems	WHO simple eye chart[5, 31, 32]<br>Binocular uncorrected visual acuity[49]<br>Weber and Rinne test[5]<br>Community Health Record Management System (CHRMS)[17]

Abbreviations: ICOPE=Integrated Care of Older People.

In this review, for ease of categorizing studies based on measurement tools, we designated the ICOPE step 2 assessment tool to include only the most widely utilized measurement tools for each subdomain of intrinsic capacity among the included studies.

Supplementary Table 2 Meta-regression analyses result.

Factors	β	SE	*Z*-score	*P*-value	95% CI
Economic status of countries	0.14	0.08	1.65	0.099	-0.03-0.30
Stage of ageing society of countries	0.19	0.09	2.23	0.026	0.02-0.36
Setting of data collection	-0.01	0.02	-0.27	0.789	-0.05-0.04
Intrinsic capacity measurement tool	0.06	0.04	1.55	0.120	-0.02-0.15

Abbreviations: CI: confidence intervals.

Supplementary Table 3 Methodological quality of the 56 included studies.

Author, year	JBI (Joanna Briggs Institute) Critical Appraisal Tools								
	Item1	Item2	Item3	Item4	Item5	Item6	Item7	Itrm8	Item9
Chang et al., 2023 [1]	Yes	No	Yes	Yes	Yes	Yes	Yes	Yes	Yes
Chen et al., 2023 [2]	Yes	No	Yes	Yes	Yes	Yes	Unclear	Yes	Unclear
Cheng et al., 2021 [3]	Yes	No	Yes	Yes	Yes	Yes	Unclear	Yes	Unclear
García-Chanes et al., 2022 [50]	Yes	Yes	Yes	Yes	Yes	Yes	Yes	Yes	No
Gaussens et al., 2023 [4]	Yes	No	Yes	Yes	Yes	Yes	Yes	Yes	Unclear
González-Bautista et al., 2021 [51]	Yes	No	Yes	Yes	Yes	Yes	Yes	Yes	Unclear
Gonzalez-Bautista et al., 2023 [35]	Yes	Yes	Yes	Yes	Yes	Yes	Yes	Yes	Unclear
Gutiérrez-Robledo et al., 2021 [52]	Yes	Yes	Yes	Yes	Yes	Yes	Yes	Yes	Unclear
Huang et al., 2022 [15]	Yes	No	No	Yes	Yes	Yes	Yes	Yes	Unclear
Jia et al., 2023 [53]	Yes	Yes	Yes	Yes	Yes	Yes	Yes	Yes	Yes
Jiang et al., 2023a [16]	Yes	Yes	Yes	Yes	Yes	Yes	Yes	Yes	Yes
Jiang et al., 2023b [32]	Yes	No	Yes	Yes	Yes	Yes	Unclear	Yes	Unclear
Jiang et al., 2023c [17]	Yes	Yes	Yes	Yes	Yes	Yes	Yes	Yes	Yes
Leung et al., 2022 [5]	Yes	No	Yes	Yes	Yes	Yes	Yes	Yes	Unclear
Li et al., 2021 [18]	Yes	No	No	Yes	Yes	Yes	Unclear	Yes	Unclear

Lin et al., 2022 [33]	Yes	No	Yes	Yes	Yes	Yes	Unclear	Yes	Unclear
Lin et al., 2023 [34]	Yes	Yes	Yes	Yes	Yes	Yes	Yes	Yes	Unclear
Liu et al., 2021a [19]	Yes	No	Yes	Yes	Yes	Yes	Unclear	Yes	Unclear
Liu et al., 2021b [6]	Yes	No	Yes	Yes	Yes	Yes	Yes	Yes	Unclear
Liu et al., 2022 [54]	Yes	No	Yes	Yes	Yes	Yes	Unclear	Yes	Unclear
Lu et al., 2023 [20]	Yes	No	No	Yes	Yes	Yes	Unclear	Yes	Yes
Ma et al., 2020 [8]	Yes	No	Yes	Yes	Yes	Yes	Yes	Yes	Unclear
Ma et al., 2021a [7]	Yes	No	Yes	No	Yes	Yes	Unclear	Yes	Unclear
Ma et al., 2021b [45]	Yes	Unclear	No	Yes	Yes	Yes	Unclear	Yes	Unclear
Ma et al., 2021c [47]	Yes	Yes	Yes	Yes	Yes	Yes	Yes	Yes	No
Ma et al., 2023 [21]	Yes	No	Yes	Yes	Yes	Yes	Yes	Yes	Unclear
Mathur et al., 2022 [9]	Yes	No	Yes	Yes	Yes	Yes	Unclear	Yes	Unclear
Meng et al., 2022 [41]	Yes	No	Yes	Yes	Yes	Yes	Unclear	Yes	Unclear
Merchant et al., 2022 [31]	Yes	No	Yes	Yes	Yes	Yes	Yes	Yes	Unclear
Muneera et al., 2023 [55]	Yes	Yes	Yes	Yes	Yes	Yes	Yes	Yes	Yes
Nagae et al., 2023 [56]	Yes	No	Yes	Yes	Yes	Yes	Yes	Yes	Unclear
Pagès et al., 2022 [10]	Yes	No	Yes	Yes	Yes	Yes	Yes	Yes	Unclear
Plácido et al., 2023 [39]	Yes	Yes	Yes	Yes	Yes	Yes	Yes	Yes	Yes
Prince et al., 2021 [36]	Yes	Yes	Yes	Yes	Yes	Yes	Yes	Yes	Yes
Rarajam Rao et al., 2023 [40]	Yes	Yes	Yes	Yes	Yes	Yes	Yes	Yes	Unclear
Rojano et al., 2023 [11]	Yes	No	No	Yes	Yes	Yes	Unclear	Yes	No
Saiyare et al., 2023 [12]	Yes	Yes	Yes	Yes	Yes	Yes	Yes	Yes	Unclear
Shi et al., 2023 [22]	Yes	No	Yes	Yes	Yes	Yes	Yes	Yes	Unclear
Sun et al., 2022 [23]	Yes	No	No	Yes	Yes	Yes	Yes	Yes	Unclear
Tang et al., 2023 [13]	Yes	No	Yes	Yes	Yes	Yes	Unclear	Yes	Unclear
Tavassoli et al., 2022 [24]	Yes	No	Yes	Yes	Yes	Yes	Yes	Yes	Unclear
Tay et al., 2023 [25]	Yes	No	Yes	Yes	Yes	Yes	Unclear	Yes	No
Wang et al., 2022 [48]	Yes	No	No	Yes	Yes	Yes	Yes	Yes	Yes
Wu et al., 2022 [46]	Yes	No	Yes	Yes	Yes	Yes	Unclear	Yes	Unclear
Yang et al., 2023 [42]	Yes	No	Yes	Yes	Yes	Yes	Unclear	Yes	Unclear
You et al., 2023 [44]	Yes	No	No	Yes	Yes	Yes	Unclear	Yes	Unclear
Yu et al., 2021 [14]	Yes	Yes	Yes	Yes	Yes	Yes	Yes	Yes	No
Yu et al., 2022 [37]	Yes	Yes	Yes	Yes	Yes	Yes	Yes	Yes	Unclear
Zhang et al., 2020a [26]	Yes	No	No	Yes	Yes	Yes	Yes	Yes	Unclear
Zhang et al., 2020b [27]	Yes	No	No	Yes	Yes	Yes	Yes	Yes	Unclear
Zhang et al., 2023a [28]	Yes	No	No	Yes	Yes	Yes	Yes	Yes	Unclear
Zhang et al., 2023b [49]	Yes	No	No	Yes	Yes	Yes	Unclear	Yes	Unclear
Zhang et al., 2023c [38]	Yes	Yes	Yes	Yes	Yes	Yes	Yes	Yes	Yes
Zhao et al., 2021 [43]	Yes	Yes	Yes	Yes	Yes	Yes	Yes	Yes	No
Zhao et al., 2023 [30]	Yes	No	Yes	Yes	Yes	Yes	Yes	Yes	Yes
Zhu et al., 2023 [29]	Yes	No	Yes	Yes	Yes	Yes	Unclear	Yes	Unclear

Item1: Was the sample frame appropriate to address the target population?

Item2: Were study participants sampled in an appropriate way?
Item3: Was the sample size adequate?
Item4: Were the study subjects and the setting described in detail?
Item5: Was the data analysis conducted with sufficient coverage of the identified sample?
Item6: Were valid methods used for the identification of the condition?
Item7: Was the condition measured in a standard, reliable way for all participants?
Item8: Was there appropriate statistical analysis?
Item9: Was the response rate adequate, and if not, was the low response rate managed appropriately?

References

1. Chang YH, Chen YC, Ku LE, Chou YT, Chen HY, Su HC, Liu CH, Wu YL, Cheng HJ, Yang YC *et al*: **Association between sleep health and intrinsic capacity among older adults in Taiwan**. *Sleep medicine* 2023, **109**:98-103.
2. Chen ZJ, Tang FP, Chang SY, Chung HL, Tsai WH, Chou SS, Yeh HC, Tung HH: **Resilience-happiness nexus in community-dwelling middle-aged and older adults: Results from Gan-Dau Healthy Longevity Plan**. *Archives of gerontology and geriatrics* 2023, **116**:105162.
3. Cheng YC, Kuo YC, Chang PC, Li YC, Huang WT, Chen W, Chou CY: **Geriatric Functional Impairment Using the Integrated Care for Older People (ICOPE) Approach in Community-Dwelling Elderly and Its Association with Dyslipidemia**. *Vascular health and risk management* 2021, **17**:389-394.
4. Gaussens L, González-Bautista E, Bonnefoy M, Briand M, Tavassoli N, De Souto Barreto P, Rolland Y, On Behalf Of The Gegn G: **Associations between Vitality/Nutrition and the Other Domains of Intrinsic Capacity Based on Data from the INSPIRE ICOPE-Care Program**. *Nutrients* 2023, **15**(7).
5. Leung AYM, Su JJ, Lee ESH, Fung JTS, Molassiotis A: **Intrinsic capacity of older people in the community using WHO Integrated Care for Older People (ICOPE) framework: a cross-sectional study**. *BMC geriatrics* 2022, **22**(1):304.
6. Liu S, Yu X, Wang X, Li J, Jiang S, Kang L, Liu X: **Intrinsic Capacity predicts adverse outcomes using Integrated Care for Older People screening tool in a senior community in Beijing**. *Archives of gerontology and geriatrics* 2021b, **94**:104358.
7. Ma L, Zhang Y, Liu P, Li S, Li Y, Ji T, Zhang L, Chhetri JK, Li Y: **Plasma N-Terminal Pro-B-Type Natriuretic Peptide Is Associated with Intrinsic Capacity Decline in an Older Population**. *The journal of nutrition, health & aging* 2021a, **25**(2):271-277.
8. Ma L, Chhetri JK, Zhang Y, Liu P, Chen Y, Li Y, Chan P: **Integrated Care for Older People Screening Tool for Measuring Intrinsic Capacity: Preliminary Findings From ICOPE Pilot in China**. *Frontiers in medicine* 2020, **7**:576079.
9. Mathur A, Bhardwaj P, Joshi NK, Jain YK, Singh K: **Intrinsic capacity of rural elderly in thar desert using world health organization integrated care for older persons screening tool: A pilot study**. *Indian journal of public health* 2022, **66**(3):337-340.
10. Pagès A, Costa N, González-Bautista E, Mounié M, Juillard-Condat B, Molinier L, Cestac P, Rolland Y, Vellas B, De Souto Barreto P: **Screening for deficits on intrinsic capacity domains and associated healthcare costs**. *Archives of gerontology and geriatrics* 2022, **100**:104654.
11. Rojano ILX, Blancafort-Alias S, Prat Casanovas S, Forné S, Martín Vergara N, Fabregat Povill P, Vila Royo M, Serrano R, Sanchez-Rodriguez D, Vílchez Saldaña M *et al*: **Identification of decreased**

intrinsic capacity: Performance of diagnostic measures of the ICOPE Screening tool in community dwelling older people in the VIMCI study. *BMC geriatrics* 2023, **23**(1):106.

12. Saiyare X, Zhuoya M, LI Y, XIANG H, WANG H: **Analysis of intrinsic capacity and its influencing factors in community-dwelling elderly adults in Xinjiang Uyghur Autonomous Region**. *Chin Prev Med* 2023, **24**(10):1074-1079.
13. Tang WH, Yu TH, Lee HL, Lee YJ: **Interactive effects of intrinsic capacity and obesity on the KDIGO chronic kidney disease risk classification in older patients with type 2 diabetes mellitus**. *Diabetology & metabolic syndrome* 2023, **15**(1):1.
14. Yu J, Si H, Qiao X, Jin Y, Ji L, Liu Q, Bian Y, Wang W, Wang C: **Predictive value of intrinsic capacity on adverse outcomes among community-dwelling older adults**. *Geriatric nursing (New York, NY)* 2021, **42**(6):1257-1263.
15. Huang B, Luo T, Jiang X: **Correlation between decline in intrinsic capacity and blood pressure variability in elderly patients with hypertensive**. *Chin J Geriatr Heart Brain Vessel Dis* 2022, **24**:709-712.
16. Jiang X, Ma X, Chen F, Yang M, Zhang X, Yang X, Yan P: **Correlation between intrinsic capacity and quality of life in 1 042 community elderly people in Urumqi**. *Journal of Xinjiang Medical University* 2023a, **46**(4):561-566.
17. Jiang X, Chen F, Yang X, Yang M, Zhang X, Ma X, Yan P: **Effects of personal and health characteristics on the intrinsic capacity of older adults in the community: a cross-sectional study using the healthy aging framework**. *BMC geriatrics* 2023c, **23**(1):643.
18. Li M, Lin Y, Xing K: **Relationship between intrinsic ability and prognosis in elderly patients with acute coronary syndrome**. *Journal of Navy Medicine* 2021, **42**(5):583-587.
19. Liu S, Kang L, Liu X, Zhao S, Wang X, Li J, Jiang S: **Trajectory and Correlation of Intrinsic Capacity and Frailty in a Beijing Elderly Community**. *Frontiers in medicine* 2021a, **8**:751586.
20. Lu F, Liu S, Liu X, Li J, Jiang S, Sun X, Huang X, Wang X: **Comparison of the predictive value of intrinsic capacity and comorbidity on adverse health outcome in community-dwelling older adults**. *Geriatric nursing (New York, NY)* 2023, **50**:222-226.
21. Ma X, Jiang X, Chen F, Yang M, Yang X, Yan P: **Analysis of potential categories of intrinsic capabilities of community-dwelling older adults and their Influencing factors**. *Chin J Prev Contr Chron Dis* 2023, **31**(6):453-457.
22. Shi X, Ouyang X, Shen L, Shen X: **Relationship between decline of intrinsic capacity and frailty in elderly inpatients**. *Pract Geriatr* 2023, **37**(3):256-260.
23. Sun Y, Zhang J, Li H, Li J, Shi H, Shen J, Zhou J, Duan Y, Zhang D: **Analysis of the status and factors influencing the intrinsic capacity of the hospitalized elderly patients**. *Chinese Jounal of Integrative Nursing* 2022, **8**(12):43-47.
24. Tavassoli N, de Souto Barreto P, Berbon C, Mathieu C, de Kerimel J, Lafont C, Takeda C, Carrie I, Piau A, Jouffrey T *et al*: **Implementation of the WHO integrated care for older people (ICOPE) programme in clinical practice: a prospective study**. *The lancet Healthy longevity* 2022, **3**(6):e394-e404.
25. Tay L, Tay EL, Mah SM, Latib A, Koh C, Ng YS: **Association of Intrinsic Capacity with Frailty, Physical Fitness and Adverse Health Outcomes in Community-Dwelling Older Adults**. *The Journal of frailty & aging* 2023, **12**(1):7-15.
26. Zhang J, Zhang D, Wu J, Zhou J, Li H, Sun C: **Relationship between decline of intrinsic capacity and activity of daily living of elderly patients**. *Chin J Mod Nurs* 2020a, **26**(32):4466-4469.

27. Zhang D, Xi H, Qi H, Chen X, Li H, Wu J, Zhou J, Zhang J: **Correlation of intrinsic capacity decline with falls in the elderly**. *Chin J Geriatr* 2020b, **39**(10):1182-1185.
28. Zhang J, Li J, Wu J, Li C, Shen J, Wu W, Shi H, Yuan Y, Liu Y, Li H: **Correlation between intrinsic capacity and nutrition, glucose and lipid metabolism indexes in elderly inpatients**. *Chinese Journal of the Frontiers of Medical Science* 2023a, **15**(6):40-46.
29. Zhu L, Zong X, Shi X, Ouyang X: **Association between Intrinsic Capacity and Sarcopenia in Hospitalized Older Patients**. *The journal of nutrition, health & aging* 2023, **27**(7):542-549.
30. Zhao Y, Zhang L, Wu G, Zhou J, Song N: **Influence of intrinsic capacity decline on quality of life of the community elderly**. *Pract Geriatr* 2023, **37**(10):1014-1018.
31. Merchant RA, Chan YH, Aprahamian I, Morley JE: **Patterns of participation restriction among older adults at risk of falls and relationship with intrinsic capacity: A latent cluster analysis**. *Frontiers in medicine* 2022, **9**:1023879.
32. Jiang YS, Shi H, Kang YT, Shen J, Li J, Cui J, Pang J, Zhang C, Zhang J: **Impact of age-friendly living environment and intrinsic capacity on functional ability in older adults: a cross-sectional study**. *BMC geriatrics* 2023b, **23**(1):374.
33. Lin S, Wang F, Zheng J, Yuan Y, Huang F, Zhu P: **Intrinsic Capacity Declines with Elevated Homocysteine in Community-Dwelling Chinese Older Adults**. *Clinical interventions in aging* 2022, **17**:1057-1068.
34. Lin S, Huang M, Yang L, Chen S, Huang X, Zheng J, Yuan Y, Li N, Huang F, Zhu P: **Dietary diversity and overweight are associated with high intrinsic capacity among Chinese urban older adults (2020−2021)**. *Experimental gerontology* 2023, **177**.
35. Gonzalez-Bautista E, Llibre-Guerra JJ, Sosa AL, Acosta I, Andrieu S, Acosta D, Llibre-Rodríguez JJ, Prina M: **Exploring the natural history of intrinsic capacity impairments: longitudinal patterns in the 10/66 study**. *Age and ageing* 2023, **52**(7).
36. Prince MJ, Acosta D, Guerra M, Huang Y, Jacob KS, Jimenez-Velazquez IZ, Jotheeswaran AT, Llibre Rodriguez JJ, Salas A, Sosa AL *et al*: **Intrinsic capacity and its associations with incident dependence and mortality in 10/66 Dementia Research Group studies in Latin America, India, and China: A population-based cohort study**. *PLoS medicine* 2021, **18**(9):e1003097.
37. Yu R, Leung G, Leung J, Cheng C, Kong S, Tam LY, Woo J: **Prevalence and Distribution of Intrinsic Capacity and Its Associations with Health Outcomes in Older People: The Jockey Club Community eHealth Care Project in Hong Kong**. *The Journal of frailty & aging* 2022, **11**(3):302-308.
38. Zhang N, Zhang H, Sun MZ, Zhu YS, Shi GP, Wang ZD, Wang JC, Wang XF: **Intrinsic capacity and 5-year late-life functional ability trajectories of Chinese older population using ICOPE tool: the Rugao Longevity and Ageing Study**. *Aging clinical and experimental research* 2023c, **35**(10):2061-2068.
39. Plácido J, Marinho V, Ferreira JV, Teixeira IA, Costa EC, Deslandes AC: **Association among race/color, gender, and intrinsic capacity: results from the ELSI-Brazil study**. *Revista de saude publica* 2023, **57**:29.
40. Rarajam Rao A, Waris M, Saini M, Thakral M, Hegde K, Bhagwasia M, Adikari P: **Prevalence and Factors Associated with Impairment in Intrinsic Capacity among Community-Dwelling Older Adults: An Observational Study from South India**. *Current gerontology and geriatrics research* 2023, **2023**:4386415.
41. Meng LC, Hsiao FY, Huang ST, Lu WH, Peng LN, Chen LK: **Intrinsic Capacity Impairment Patterns and their Associations with Unfavorable Medication Utilization: A Nationwide Population-Based**

Study of 37,993 Community-Dwelling Older Adults. *The journal of nutrition, health & aging* 2022, **26**(10):918-925.
42. Yang Y, Shen S, Zeng X, Wang Y, Chen L, Chen X: **Impact of intrinsic capacity on predicting future falls and readmission in older patients**. *Chin J Geriatr* 2023, **42**(2):165-168.
43. Zhao J, Chhetri JK, Chang Y, Zheng Z, Ma L, Chan P: **Intrinsic Capacity vs. Multimorbidity: A Function-Centered Construct Predicts Disability Better Than a Disease-Based Approach in a Community-Dwelling Older Population Cohort**. *Frontiers in medicine* 2021, **8**:753295.
44. You L, Xu W, Qi J, Fu T, Chen Z: **Correlation analysis of fall events and intrinsic capacity in elderly hospital inpatients**. *Journal of Qilu Nursing* 2023, **29**(1):112-115.
45. Ma L, Liu P, Zhang Y, Sha G, Zhang L, Li Y: **High Serum Tumor Necrosis Factor Receptor 1 Levels Are Related to Risk of Low Intrinsic Capacity in Elderly Adults**. *The journal of nutrition, health & aging* 2021b, **25**(4):416-418.
46. Wu W, Sun L, Li H, Zhang J, Shen J, Li J, Zhou Q: **Approaching person-centered clinical practice: A cluster analysis of older inpatients utilizing the measurements of intrinsic capacity**. *Frontiers in public health* 2022, **10**:1045421.
47. Ma L, Chhetri JK, Zhang L, Sun F, Li Y, Tang Z: **Cross-sectional study examining the status of intrinsic capacity decline in community-dwelling older adults in China: prevalence, associated factors and implications for clinical care**. *BMJ open* 2021c, **11**(1):e043062.
48. Wang H, Zhang J, Li J, Li H, Wu J, Shen J, Wu W, Yuan Y: **Analysis of intrinsic capacity and influencing factors in community-dwelling elderly people**. *Chinese Journal of Geriatrics* 2022, **41**(5):591-595.
49. Zhang R, Guo J, Wang Q, Li B, Zhao X, Yang Y, Dong L, Li S, Tian R: **Influencing factors of intrinsic capacity decline in elderly patients with chronic non-communicable diseases**. *J Chin Pract Diagn Ther* 2023b, **37**(4):383-388.
50. García-Chanes RE, Gutiérrez-Robledo LM, Álvarez-Cisneros T, Roa-Rojas P: **Predictors of Successful Memory Aging in Older Mexican Adults**. *Behavioural neurology* 2022, **2022**:9045290.
51. González-Bautista E, de Souto Barreto P, Andrieu S, Rolland Y, Vellas B: **Screening for intrinsic capacity impairments as markers of increased risk of frailty and disability in the context of integrated care for older people: Secondary analysis of MAPT**. *Maturitas* 2021, **150**:1-6.
52. Gutiérrez-Robledo LM, García-Chanes RE, Pérez-Zepeda MU: **Screening intrinsic capacity and its epidemiological characterization: a secondary analysis of the Mexican Health and Aging Study**. *Revista panamericana de salud publica = Pan American journal of public health* 2021, **45**:e121.
53. Jia S, Zhao W, Ge M, Xia X, Hu F, Hao Q, Zhang Y, Yang M, Yue J, Dong B: **Associations between transitions of intrinsic capacity and frailty status, and 3-year disability**. *BMC geriatrics* 2023, **23**(1):96.
54. Liu Y, Ouyang J, Hu J: **Influence of aging on intrinsic ability of elderly patients and analysis of related factors**. *Chin J Clin Healthc* 2022, **25**:460-467.
55. Muneera K, Muhammad T, Pai M, Ahmed W, Althaf S: **Associations between intrinsic capacity, functional difficulty, and fall outcomes among older adults in India**. *Sci Rep* 2023, **13**(1):9829.
56. Nagae M, Umegaki H, Komiya H, Nakashima H, Fujisawa C, Watanabe K, Yamada Y, Miyahara S: **Intrinsic capacity in acutely hospitalized older adults**. *Experimental gerontology* 2023, **179**:112247.
